# Relationship between Employment Type and Self-Rated Health among Korean Immigrants in the US: Focusing on Gender and Number of Years in the US

**DOI:** 10.3390/ijerph18041654

**Published:** 2021-02-09

**Authors:** Sou Hyun Jang

**Affiliations:** Department of Sociology & Convergence Program for Social Innovation, Sungkyunkwan University, Seoul 03063, Korea; souhyunjang@skku.edu

**Keywords:** Korean immigrants, self-rated health, employment type, gender, assimilation

## Abstract

Although Korean immigrants report worse self-rated health and a higher self-employment rate than other Asian immigrant groups, the relationship between their employment type and self-rated health is understudied. This study examines the relationship between employment type and self-rated health among Korean immigrants in the US. Survey data of 421 first-generation working-age (18–64 years old) Korean immigrants in the New York–New Jersey area were analyzed. The self-administrated survey questionnaire included 39 items (e.g., sociodemographic characteristics, self-rated health, and health insurance status). A logistic regression analysis was conducted to examine the relationship between the dependent variable—self-rated health (e.g., bad/not bad vs. good/very good)—and independent variable—employment type (e.g., work at non-ethnic firms, work at co-ethnic firms, self-employed, and unemployed)—by focusing on differences regarding gender and number of years living in the US. Self-employed and unemployed Korean immigrants were less likely to report good health compared to those working in non-ethnic firms. After controlling for sociodemographic characteristics (age, gender, marital status, education, health insurance status, membership in any Koran association, religion, and English proficiency), the relationship between employment type and self-rated health remained significant among female and recent Korean immigrants. More worksite interventions by occupational health nurses that target self-employed Korean immigrants, especially women and recent immigrants, are necessary.

## 1. Introduction

Self-rated health is a subjective measure of an individual’s perception of their own health, whereas objective health status is based on the diagnosis by doctors or laboratory parameters [1]. Previous studies have found that self-rated health, which is consistent with the objective health status [1], is a valid measure [2] across different sociodemographic groups [3]. In addition to the disparities in self-rated health by age [4], gender [5], socioeconomic status [6], and place of birth [7], previous research also indicates that people who are employed tend to report better self-rated health than those who are unemployed, because employment could provide benefits, such as income, social support, and social and personal identity [8]. 

Prior studies have found that employees’ self-rated health also differs depending on employment type, which can be categorized as follows: permanent vs. non-permanent [9], precarious vs. non-precarious [10], and self-employed vs. employed [11,12]. For example, those in precarious [10] and nonstandard employment situations [13] tend not to report good health or greater health risks compared to those with full-time permanent employment. The findings regarding the relationship between self-employment and self-rated health are inconsistent; although most studies have found a negative relationship between self-employment and health [14,15], few report a positive relationship between self-employment and health status [16]. A person’s level of independence—which is the nature of self-employment—may positively influence health [17]. Yet, self-employed immigrants may need to be distinguished from self-employed natives because the former are often limited to the ethnic economy of their destination country—which is typically a secondary labor market prone to overwork and exposure to physical danger [18]—which could also provide alternative economic opportunities with ethnic resources and networks [19]. Self-employed immigrants who run their own businesses, as well as employees at co-ethnic firms with co-ethnic owners, often confront worse working conditions and low returns [20], which may negatively influence their health. Although self-employed immigrants and immigrant employees at co-ethnic firms have often been lumped together as employees in the ethnic economy, the health status of these two groups should be examined separately due to differences in their socioeconomic status [21].

A total of 1.9 million Koreans reside in the United States (US), which comprised the fifth largest Asian immigrant group as of 2017 [22]. Following Los Angeles, the New York–New Jersey region is home to the largest population of Korean immigrants [23]. Korean immigrants are known for their higher self-employment rates compared to other immigrant groups in the US [24,25,26]. For example, according to a sample from the 2007–2011 American Community Survey, Korean immigrants show higher self-employment rates (19.4%) than Vietnamese (12.9%), Chinese (8.2%), Asian Indians (6.5%), and Filipinos (4.3%) [26]. In addition to a high rate of self-employment, high numbers of Korean immigrants are also employed in an ethnic economy with co-ethnic employers or coworkers [27].

Korean immigrants report worse health than most other immigrant groups in the US [28,29]. Based on the 2016 California Health Interview Survey, 27.2% of Korean immigrants reported excellent or very good health, which was lower than the average of Chinese (41.9%), Filipino (40.8%), and Asian immigrants (35.9%) [29]. However, little research has been done on whether Korean immigrants’ employment type, including self-employment and employment at co-ethnic firms, is related to their self-rated health. A recent study [8] examined whether Korean immigrants’ employment status was related to their subjective well-being, but the study did not expand the categorization of employment type beyond employed versus unemployed status. However, some immigrants tend to be limited to the ethnic economy, as they either run their own business or work at co-ethnic firms in the US [30]. Furthermore, although female [31] and recent immigrants [32] tend to report worse health compared to male and non-recent immigrants, the understanding regarding whether female and recent immigrants are doubly disadvantaged by their employment type is limited. Given the high concentration of Korean immigrants in the New York–New Jersey area [23], which is home to a large number of self-employed Korean immigrants [33], this geographic locale may constitute an ideal research area in which the following hypotheses may be evaluated:

**Hypothesis 1** **(H1).** *Unemployed Korean immigrants are less likely to report good health than their employed counterparts.*

**Hypothesis 2** **(H2).** *Among employed Korean immigrants, those who work at co-ethnic firms and are self-employed are less likely to report good health than those who work at non-ethnic firms or for the government.*

**Hypothesis 3-a** **(H3-a).** *The relationship between employment type and self-rated health is stronger among women than among men.*

**Hypothesis 3-b** **(H3-b).** *The relationship between employment type and self-rated health is stronger among recent than among non-recent Korean immigrants.*

By analyzing the survey data of 421 working-age (18–64 years old) Korean immigrants in the New York–New Jersey area, this study had three aims as follows: (1) to compare self-rated health status among Korean immigrants based on employment status and type; (2) to examine the relationship between employment type and self-rated health; and (3) to demonstrate whether this relationship differs by gender and the number of years spent in the US. 

## 2. Theoretical Concepts and Literature Review

### 2.1. Acculturation and Assimilation 

In 1936, Redfield et al. [34] defined acculturation as “phenomena which result when groups of individuals having different cultures come into continuous first-hand contact, which subsequent changes in the original cultural patterns of either or both groups (p. 149).” Based on this definition, scholars later [35,36] suggested that acculturation could be classified into four different types (assimilation, separation, integration, and marginalization) based on the following two conditions: (1) maintaining/abandoning one’s origin culture and (2) embracing/rejecting one’s new culture. On the one hand, assimilation and marginalization commonly perceive immigrants as abandoning the culture of their home country; assimilation suggests that immigrants embrace the culture of their destination country while marginalization suggests that immigrants fail to do so. On the other hand, separation and integration suggest that immigrants will maintain the culture of their home country; however, the separation perspective suggests that immigrants will not acquire the new culture of their destination country while that of integration suggests the opposite.

In summary, while assimilation is a one-way process referring to the phenomenon in which immigrants become a part of the dominant culture in their destination country by incorporating their new culture and losing their original culture [36,37], acculturation is a broader concept and could be a bidirectional process leading to changes in the cultures of both immigrants and their destination country, as the definition above describes and as was previously indicated [38]. Immigrants’ adaptation is not a simple process and is instead complex, with many possible different pathways for each individual and group, none of which are easily categorized. As previously noted [39], the suitability of an acculturation strategy among immigrants might depend on the culture of origin, its interaction with the host culture, or other contexts.

Often, acculturation and assimilation have been used interchangeably in discussions on this process; for example, structural acculturation is also termed social assimilation [40]. Yet, since this study aims to examine the relationship between Korean immigrants’ employment type and self-rated health without considering changes in US society, the concept of assimilation is more suitable than acculturation for this study. Furthermore, earlier studies have mentioned that Korean immigrants have acknowledged “the necessity of assimilation” into the US society [41], although they tend to experience a slower assimilation than other Asian immigrants in the US [42]. In particular, Korean immigrants engaged in the ethnic economy as entrepreneurs or workers tend to experience slower assimilation into US society than Korean immigrants working in the general labor market due to structural disadvantages (e.g., maintaining ethnic attachment by meeting Korean-speaking customers) that impede assimilation [42].

### 2.2. Number of Years in Host Country and Employment

Immigrants tend to experience difficulties in the labor market when they migrate to their new country due to a language barrier, the undervaluation of human capital, discrimination in the labor market of the host country, etc. [43,44,45]. Nonetheless, they tend to achieve labor market assimilation, as they spend more years in the host country. For example, immigrants report a lower rate of employment compared to their native-born counterparts, but their employment rate increases as the duration of their stay in the US also increases. This occurs because they acquire more knowledge of the labor market in their host country, as well as English proficiency, although they do seemingly reach a point of stagnation later [44,46]. Other labor market characteristics, such as occupational attainment and earnings, also follow a similar pattern [43,47]; immigrants experience downgraded mobility, but later rebound. 

Compared to other labor market characteristics, the relationship between the duration of stay in the host country and employment type has been understudied. A few studies have found that a longer duration in a host country is positively related to higher propensity for self-employment among immigrants in Canada [48], and other Western countries [49]. In addition to self-employment, the current study attempts to include other employment types (i.e., work at non-ethnic firms, work at co-ethnic firms, and unemployed) to examine its relationship with the self-rated health of Korean immigrants in the US among immigrants by their years of stay in the US. 

### 2.3. Number of Years in Host Country and Self-Rated Health 

In general, previous studies have found that a longer duration in a host country is negatively related to health status among immigrants in many host countries, such as the US [32,50,51], Canada [52], and Australia [53]. In other words, overall, recent immigrants tend to report better self-rated health, while this effect decreases as the length of their stay in the US increases. Despite a large volume of earlier studies that have examined the relationship between the duration of stay in a host country and self-rated health among immigrants, most did not distinguish ethnicity among the racial/ethnic groups. Asian immigrants are not homogeneous and each group has its own specific culture and path to assimilation that could impact health [54]. Furthermore, the overall landscape of health in the home country might be related to health among immigrants. For example, Koreans in South Korea are known to have worse self-rated health as they get older with the effect of different socioeconomic status [55,56]. Thus, in the multivariate analyses of the current study, Korean immigrant participants’ age was controlled when the relationship was between employment type and self-rated health.

## 3. Materials and Methods

### 3.1. Data 

To examine the relationship between employment type and self-rated health among Korean immigrants, this study used a quantitative research method; it analyzed the survey data of Korean immigrants in the New York–New Jersey area. The original data, which were used in the author’s dissertation regarding Korean immigrants’ medical tourism to their home country, included 507 first-generation Korean immigrant adults who met the following criteria: identified as Korean, were born outside of the US, were aged ≥18 years, and migrated to the country when they were aged ≥13 years. Because the focus of the current study is the relationship between employment type and self-rated health, Korean immigrants who were 65 years old and older and were presumably retired were excluded from the current study. Consequently, the sample included working-age Korean immigrant survey respondents who were 18–64 years old at the time of the survey (*n* = 421).

A self-administrated survey questionnaire was developed by the author. Most survey questions were adapted based on the measures of the New Immigrant Survey (NIS) [57]. The survey questionnaire, which originally attempted to examine Korean immigrants’ utilization of healthcare and medical tourism, had 39 items, including sociodemographic characteristics (e.g., gender, age, number of years in the US, area of residence, educational level, English proficiency, marital status, occupation, employment status and type, religion, membership in Korean association), health status (e.g., self-rated health), health insurance status (e.g., insured status and type), healthcare utilization (e.g., having a family doctor, number of doctor visits in the last five years, and barriers to utilization of US healthcare), transnational ties with one’s home country (e.g., contacting or visiting one’s home country) and medical tourism to one’s home country (e.g., the frequency, type, reasons, and level of satisfaction with medical care received in Korea). 

The Korean immigrant survey participants were recruited between fall 2013 and spring 2014 at three ethnic community centers, the Chuseok Moon Festival, four Korean Protestant churches, two Catholic churches, one Buddhist temple, and one Won Buddhist temple in the New York–New Jersey metropolitan area. After receiving permission from the directors of the ethnic community centers, the organizers of the festival, and the pastors of the churches and temples, the author distributed the survey questionnaire to participants in person. Before each potential survey participant began filling out the questionnaire, the author confirmed their age and date of arrival in the US to filter out those who did not meet the inclusion criteria. Respondents completed the self-administered survey in approximately 20–30 min. Although participants gave valid answers for most questions, a value was missing for the question that asked about respondents’ legal status; only 264 respondents (52.07%) gave an answer regarding their legal status. Due to these missing values, Korean immigrant respondents’ legal status was not included in the current study. 

Two versions of the survey questionnaire (one in Korean and the other in English) were available, but all survey participants chose the questionnaire in Korean. The translation did not follow the guidelines of the International Test Commission [58]. However, the translation was conducted by an author who is a bilingual Korean who is familiar with both languages and cultures, as emphasized in the guidelines. The validity of the translated survey questionnaire between the two languages was confirmed by the Institutional Review Board (IRB) at the City University of New York (CUNY). No financial reward was given for participation in the study. 

### 3.2. Measurements 

Most variables were adapted from the measurements of the NIS data. The dependent variable of this study was self-rated health. The participants were asked to report their self-rated health by completing the following question, “In general, would you say your health is…?” with one of the possible responses as follows: (1) bad, (2) not bad, (3) good, and (4) very good. Previous studies have confirmed that a similar question with four (e.g., very poor, poor, good, and very good; [2]) or five possible answers (e.g., poor, fair, good, very good, and excellent; [59]) is a valid measure of self-rated health among Korean immigrants. For the logistic regression analysis, the first and last two answers were combined: bad/not bad (reference group) vs. well/very well. 

The independent variable, employment type, was categorized based on the following question: “If you have an occupation, in which of the following categories would you classify it?” Possible answers included non-Korean (non-ethnic)-owned firms, government or public sector, Korean (co-ethnic)-owned firms, self-employed, and not applicable (student, retired, unemployed). These answers were recoded as (1) non-ethnic firms or government job (reference group), who are not in the ethnic economy, (2) co-ethnic firms, (3) self-employed, and (4) unemployed, including people in the labor force without any occupation and those not in the labor force (e.g., student or retired). 

Control variables were chosen and included in the statistical model based on the findings of previous studies, which found a significant correlation between self-rated health and age, gender, marital status, and education [60,61,62]; social capital [2]; number of years in the US [32]; and health insurance [63]. In the current study, the following control variables were included: age groups (18–29, 30–39, 40–49, and 50–64 years); gender (male vs. female); marital status (unmarried vs. married); educational attainment (high school or below, some college, BA degree, graduate school); and health insurance status (no vs. yes). Variables were also included to measure Korean immigrants’ social capital: membership in any Korean association, such as alumni, business, or international student associations; Korean community organization (no vs. yes); and religious affiliation (none, Protestant, Catholic, Buddhist/other). 

To measure immigrant assimilation, researchers have suggested evaluation that considers different aspects, such as economy, culture, space, marriage status, and civic life. [64,65]. Due to data limitation, in the current study, two measures of assimilation (English proficiency; not well/a little vs. well/very well and the number of years in the US; <10 years vs. ≥10 years) were used. As previously indicated [65], most other measures of assimilation tend to increase as immigrants live in the US for a longer period.

### 3.3. Data Analysis 

First, the Chi-squared test was used to examine the differences among Korean immigrants by employment type. Second, a logistic regression was conducted to examine whether employment type is related to self-rated health. Third, the logistic regression analysis models included control variables to determine whether factors other than employment type are related to self-rated health among Korean immigrant participants. Each logistic regression analysis was carried out using different subgroups based on gender and number of years in the US. All statistical analyses were conducted using Stata 13.0. The significance level was set at *p* < 0.05. 

### 3.4. Ethnical Considerations

This study received approval with a waiver of the general requirements for obtaining written consent by the IRB at CUNY. Verbal consent was obtained from all subjects involved in the study (IRB No. 491073-2).

## 4. Results

### 4.1. Participant Characteristics

Table 1 presents the characteristics of survey participants by employment type. Among working-age Korean immigrant participants (*n* = 421), 28.5% were unemployed, while 71.5% were employed. Employment in co-ethnic firms was the most popular employment type (39.2%), followed by self-employment (18.3%), and employment in non-ethnic firms or governmental organizations (14.0%). Approximately one-fifth (21.4%) were 18–29 years old, approximately a quarter (23.0%) were 30–39, and 27.8% were 40–49 and 50–64, respectively. Just over half of the participants (57.5%) were women and married (62.0%). Most participants had a high level of education; about two-thirds had a BA or higher. Approximately one-third (67.5%) were insured. Just over half (58.2%) were members of a Korean association (e.g., alumni, business, or international student associations; Korean community organizations). The vast majority (95.5%) had religious affiliations, and Protestantism was the most popular, followed by Catholicism and Buddhism. Over one-quarter of the participants (27.1%) were new immigrants who have lived in the US for fewer than 10 years, whereas the remaining (72.9%) have lived in the US for 10 years or longer. Regarding English proficiency, about half (48.5%) spoke English either not well or a little, while the other half (51.5%) spoke English either well or very well. 

Several characteristics differed by employment type among Korean immigrant participants: age, gender, marital status, education, health insurance status, number of years in the US, and English proficiency. Participants who worked at non-ethnic firms or for the government tended to be more educated, better insured, and had better English proficiency than self-employed Korean immigrants or those working in an ethnic economy. Furthermore, self-employed Korean immigrants tended to be older (50–64 years old), male, married, and with lower educational attainment, a lower rate of health insurance, and lower English proficiency compared to participants engaged in other types of employment. Korean immigrants working at co-ethnic firms were likely to be 30–49 years old, married, less insured, and non-recent immigrants who have lived in the US for 10 years or longer. Unemployed Korean immigrant participants tended to be young (18–29 years old), female, unmarried, and recent immigrants who have lived in the US for fewer than 10 years. 

### 4.2. Self-Rated Health by Employment Type

**Figure 1:**
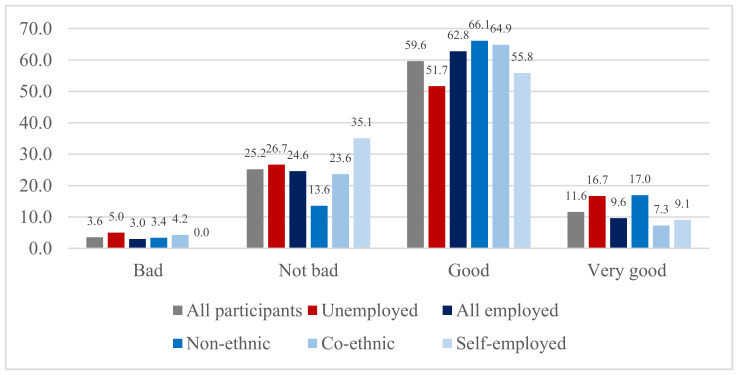
Self-rated Health by Employment Type (%).

Figure 1 compares self-rated health by Korean immigrants’ employment type. This relationship between self-rated health and employment type was statistically significant among Korean immigrants based on the Chi-squared test (*p* = 0.019). On average, most participants reported good or very good self-rated health (59.6% and 11.6%, respectively), while 25.2% reported not bad self-rated health, and 3.6% reported bad self-rated health. While 72.4% of all employed Korean immigrants reported either good or very good health, 68.4% of unemployed Korean immigrants reported the same. Thus, H1 was accepted. When compared to employment type, self-employed Korean immigrants reported the lowest good or very good self-rated health (64.9%), followed by those working at co-ethnic firms (72.2%), while those working at non-Korean firms or for the government reported the highest rate, good or very good self-rated health (83.1%). Therefore, H2 was also accepted. 

### 4.3. Relationship between Self-Rated Health and Employment Type 

Table 2 shows the relationship between employment type and self-rated health among Korean immigrants by gender and number of years in the US. Among the participants, self-employed and unemployed Korean immigrants were less likely to report good or very good self-rated health (odds ratio [OR] = 0.38 and 0.44, respectively; *p* < 0.05) compared to Korean immigrants working at non-ethnic firms. Korean immigrants working at co-ethnic firms were also less likely to report good or very good health than those working at non-ethnic firms, but this relationship was not statistically significant. 

Employment type was not related to self-rated health among Korean immigrant men and those who have lived in the US for 10 years or longer. Conversely, it was significantly associated with Korean immigrant women and recent Korean immigrants who have lived in the US for 10 years or less. Therefore, H3-a and H3-b were accepted. Among Korean women, those who were self-employed or unemployed were less likely to report good or very good self-rated health (OR = 0.17 and 0.32, respectively; *p* < 0.05) than those working at non-ethnic firms. Among recent immigrants, self-employed Korean immigrants and those working at co-ethnic firms were less likely to report good or very good self-health (OR = 0.11 and 0.05, respectively; *p* < 0.05) than those working at non-ethnic firms. 

Table 3 shows whether the relationship between employment type and self-rated health remains consistent after controlling for other variables, such as age, gender, marital status, education, health insurance status, membership in a Korean association, religion, number of years lived in the US, and English proficiency. After controlling for these variables, only employment at co-ethnic firms was significantly related to self-rated health; Korean immigrants working at co-ethnic firms were less likely to report good or very good health than those working at non-ethnic firms (OR = 0.41, 95% confidence interval [CI] = 0.18–0.95). Additionally, age was negatively related to self-rated health, as Korean immigrants who were 40 years old or above had lower ORs than their younger counterparts, who were 18–29 years old. Those with a BA degree were more likely to report good or very good self-rated health than those with a high school education or less (OR = 1.90, 95% CI = 1.07–3.37).

When the analyses were carried out on subgroups based on gender and number of years in the US, employment type was not significantly related to self-rated health among men and non-recent immigrants after controlling for the previously stated variables. Instead of employment type, having a BA degree (OR = 2.07; 95% CI = 1.04–4.12) and health insurance (OR = 0.48; 95% CI = 0.25–0.95) were associated with self-rated health among non-recent Korean immigrants who have lived in the US for 10 years or longer. None of the variables significantly predicted good self-rated health among Korean immigrant men. 

Conversely, employment type remained significant among Korean immigrant women and recent Korean immigrants; self-employed and unemployed immigrants and those working at co-ethnic firms were less likely to report good or very good health than those working at non-ethnic firms in each subgroup (women and recent immigrants). Among Korean immigrant women, being over the age of 40 and a recent immigrant was negatively correlated with good self-rated health.

## 5. Discussion

The current study aimed to evaluate the relationship between employment type and self-rated health among Korean immigrants; this relationship was confirmed via an analysis of the survey data of 421 participants. Being unemployed rather than employed (H1), working at co-ethnic firms, and being self-employed rather than working at non-ethnic firms (H2) were negatively correlated with good self-rated health among Korean immigrants. These findings suggest that although the ethnic economy could provide opportunities, such as ethnic resources and networks [19], which could positively influence the health of Korean immigrants, it also might provide substandard working conditions for them compared to individuals who work in the mainstream economy in the US.

Results also showed that the significant relationship between employment type and self-rated health remained among Korean immigrants who were female and recent immigrants who have lived in the US for less than 10 years, but no such relationship was revealed among Korean men and non-recent Korean immigrants who have lived in the US for 10 years or longer (H3-a and H3-b). This finding may reflect the gender and recency of arrival inequality, as well as the specific labor market context in the ethnic enclave economy of the US. Immigrant women and recent immigrants tend to confront more difficulties in the labor market of the destination country than immigrant men and immigrants who have lived in the US for a longer period [66]. Thus, they are more likely to remain in the ethnic economy than their male and more assimilated immigrant counterparts, who have more opportunities to enter the mainstream economy [67]. As a previous study [68] noted, many Korean immigrant women are unpaid family workers in the ethnic economy. They often endure long working hours—particularly, married Korean immigrant self-employed women who have longer working hours than their husbands—in substandard working conditions that may influence the severity of their self-rated health. Although earlier studies have found that recent immigrants are likely to report better self-rated health than those who have been in the US for a longer period [32,50,51], as noted in the previous section, immigrants who have recently arrived in the US tend to have limited English proficiency and social networks in their destination country. Thus, they may be more likely to work as employees in an ethnic economy, which is associated with poor working conditions and low wages [30], therefore leading them to perceive their health as bad. Since the current study confirms that female and recent immigrants in an ethnic economy tend to report worse self-health rates than those working in the mainstream labor market in the US, more support, such as improvements in their working conditions and environment, is needed. 

It is noteworthy that none of the variables included in the statistical model significantly predict self-rated health among Korean men, who could be considered a more prestigious group in the labor market. This finding echoes that of a previous study [13], which found no impact from self-employment or nonstandard employment on health outcomes among men in Korea; however, these types of employment negatively impact health among women. To explain this difference by gender, Lim and her colleagues [13] suggested that women are already negatively selected into these types of employment. Future studies must explore other factors related to the health of Korean immigrant men in the US. 

This study has several limitations. First, a convenience sample from a restricted area has been used. Korean immigrants in other US regions may reveal different patterns in the relationship between their employment type and self-rated health. Moreover, the data were collected between 2013 and 2014, and are therefore somewhat old for use in this type of study. However, since most secondary data (e.g., the National Health Interview Survey or the National Health and Nutrition Examination Survey (NHANES) do not specify Korean ethnicity, this study still contributes to the limited literature on Korean immigrants’ health. Second, no worksite characteristics (e.g., the number of employees in the workplace, the fields of work, working hours, and working conditions), duration of time working at current job, and part-time vs. full-time status were included in this study, which could be remedied by future studies. The unobserved health or working conditions (e.g., hazards, dangers), in addition to employment type, may be an important influence on Korean immigrants’ self-rated health. Third, marital status was assessed as married vs. unmarried, so further analyses based on more diverse options in marital status, such as divorced or widowed, were not applied in the current study. Fourth, although this study found no relationship between marital status and self-rated health among Korean immigrants, family and parenting status may be related to the health of Korean immigrants, as noted by a previous study [13]. Finally, this study measured self-rated health through one simple question, rather than multiple questions, including how much a respondent paid for medicine within the past year or how many times they visited a doctor to address a health complaint. Despite the overall consistency between self-rated health and objective health status [1], the authors of future studies may wish to examine whether employment type is related to any diagnosed health conditions. Further, in addition to physical health, mental health outcomes—such as depression or stress—could be considered in the context of their relationship with employment type and could also be supported by occupational health nurses. 

Despite these limitations, this study’s findings will contribute to the limited literature on Korean immigrant health and suggest policy implications in the following two ways. First, to the best of the author’s knowledge, this is one of the first studies to examine Korean immigrants’ self-rated health by employment type and include the categories of co-ethnic firms and self-employment. Second, this work revealed disparities in self-rated health among Korean immigrants by gender and number of years in the US; female and recent immigrants employed in the ethnic economy comprise the most vulnerable groups, which require relatively more support for better health. Based on the findings of the current study, social capital, such as participation in Korean associations or religious institutions, was revealed as unrelated to health among Korean immigrants. Thus, from a policy perspective, linguistically and culturally concordant health education programs based on worksites, rather than ethnic organizations, could be a pathway towards promoting better health among Korean immigrants in the US. As Korean immigrants tend to depend heavily on co-ethnic healthcare professionals in the US [69], Korean immigrant healthcare professionals in the US with fewer linguistic and cultural barriers to communication with Korean immigrants could play a potential role in improving health among Korean immigrants and their community. 

## Figures and Tables

**Table 1:** Characteristics of Survey Participants by Employment Type, *n* (%).

	All (*n* = 421)	Non-Ethnic (*n* = 59)	Co-Ethnic (*n* = 165)	Self-Employed (*n* = 77)	Unemployed (*n* = 120)
Employment Type					
Unemployed	120 (28.5)				
Employed	301 (71.5)				
Non-ethnic	59 (14.0)				
Co-ethnic	165 (39.2)				
Self-employed	77 (18.3)				
Age (year) *					
18–29	90 (21.4)	11 (18.7)	25 (15.1)	4 (5.2)	50 (41.6)
30–39	97 (23.0)	12 (20.3)	60 (36.4)	8 (10.4)	17 (14.2)
40–49	117 (27.8)	20 (33.9)	53 (32.1)	21 (27.3)	23 (19.2)
50–64	117 (27.8)	16 (27.1)	27 (16.4)	44 (57.1)	30 (25.0)
Gender *					
Male	179 (42.5)	25 (42.4)	80 (48.5)	43 (55.8)	31 (25.8)
Female	242 (57.5)	34 (57.6)	85 (51.5)	34 (44.2)	89 (74.2)
Marital Status *					
Unmarried	156 (38.0)	25 (42.4)	55 (33.3)	15 (19.5)	61 (50.8)
Married	265 (62.0)	34 (57.6)	110 (66.7)	62 (80.5)	59 (49.2)
Education *					
High school or less	96 (22.8)	5 (8.5)	30 (18.2)	26 (33.8)	35 (29.2)
Some college	50 (11.9)	5 (8.5)	22 (13.3)	13 (16.9)	10 (8.3)
BA	208 (49.4)	31 (52.5)	92 (55.8)	29 (37.6)	56 (46.7)
Graduate school	67 (15.9)	18 (30.5)	21 (12.7)	9 (11.7)	19 (15.8)
Health Insurance *					
Uninsured	137 (32.5)	9 (15.3)	43 (26.1)	35 (45.5)	50 (41.7)
Insured	284 (67.5)	50 (84.7)	122 (73.9)	42 (54.5)	70 (58.3)
Membership in any Korean Association					
No	176 (41.8)	26 (44.1)	73 (44.2)	27 (35.1)	50 (41.7)
Yes	245 (58.2)	33 (55.9)	92 (55.8)	50 (64.9)	70 (58.3)
Religion					
None	19 (4.5)	1 (1.7)	7 (4.2)	6 (7.8)	5 (4.2)
Protestant	247 (58.7)	34 (57.7)	109 (66.1)	38 (49.3)	66 (55.0)
Catholic	87 (20.7)	12 (20.3)	27 (16.4)	18 (23.4)	30 (25.0)
Buddhist/other	68 (16.1)	12 (20.3)	22 (13.3)	15 (19.5)	19 (15.8)
Number of Years in the United States *					
<10 years	114 (27.1)	13 (22.0)	33 (20.0)	11 (14.3)	57 (47.5)
≥10 years	307 (72.9)	46 (78.0)	132 (80.0)	66 (85.7)	63 (52.5)
English Proficiency *					
Not well/a little	204 (48.5)	17 (28.8)	84 (50.9)	49 (63.6)	54 (45.0)
Well/very well	217 (51.5)	42 (71.2)	81 (49.1)	28 (36.4)	66 (55.0)

* statistically significant at *p* < 0.05 (Chi-squared test).

**Table 2:** Relationship Between Self-Rated Health and Employment Type by Gender and Number of Years in the US (OR, 95% CI).

	All	Gender		Number of Years in the US	
		Men	Women	Recent Immigrants (<10 Years)	Non-Recent Immigrants (≥10 Years)
Non-ethnic firms	1.00 (ref)	1.00 (ref)	1.00 (ref)	1.00 (ref)	1.00 (ref)
Co-ethnic firms	0.53 (0.25–1.13)	0.62 (0.21–1.85)	0.46 (0.16–1.35)	0.11 (0.01–0.97) *	0.76 (0.33–1.74)
Self-employed	0.38 (0.17–0.86) *	0.83 (0.25–2.76)	0.17 (0.05–0.55) **	0.05 (0.004–0.52) *	0.56 (0.23–1.37)
Unemployed	0.44 (0.20–0.96) *	0.86 (0.24–3.12)	0.32 (0.11–0.92) *	0.20 (0.02–1.63)	0.49 (0.20–1.19)
Cons	4.90 (2.48–9.67) ***	4.00 (1.50–10.66) **	5.80 (2.25–14.98) ***	12.00 (1.56–92.29) *	4.11 (1.98–8.52) ***
Pseudo R^2^	0.0128	0.005	0.0377	0.072	0.0097
N	421	179	242	114	307

*** *p* < 0.001; ** *p* < 0.01; * *p* < 0.05; OR, odds ratio; CI, confidence interval.

**Table 3:** Logistic Regression Predicting Self-Rated Health by Gender and Number of Years in the US (OR, 95% CI).

	All	Gender		Number of Years in the US	
		Men	Women	Recent Immigrants (<10 Years)	Non-Recent Immigrants (≥10 Years)
Employment Type					
Non-ethnic firms	1.00 (ref)	1.00 (ref)	1.00 (ref)	1.00 (ref)	1.00 (ref)
Co-ethnic firms	0.41 (0.18–0.95) *	0.50 (0.15–1.69)	0.27 (0.08–0.93) *	0.04 (0.002–0.59) *	0.61 (0.25–1.51)
Self-employed	0.42 (0.17–1.104)	1.02 (0.25–4.17)	0.15 (0.04–0.55) **	0.02 (0.001–0.40) **	0.59 (0.21–1.63)
Unemployed	0.46 (0.19–1.08)	1.39 (0.29–6.61)	0.20 (0.06–0.67) *	0.07 (0.005–0.89) *	0.46 (0.17–1.27)
Age (years)					
18–29	1.00 (ref)	1.00 (ref)	1.00 (ref)	1.00 (ref)	1.00 (ref)
30–39	1.12 (0.49–2.54)	2.17 (0.58–8.01)	0.50 (0.15–1.64)	1.02 (0.21–4.87)	1.38 (0.45–4.19)
40–49	0.37 (0.15–0.90) *	0.49 (0.11–2.21)	0.22 (0.07–0.76) *	0.45 (0.06–3.40)	0.38 (0.12–1.13)
50–64	0.36 (0.14–0.92) *	0.64 (0.13–3.11)	0.16 (0.05–0.58) **	0.18 (0.02–1.83)	0.39 (0.13–1.19)
Gender					
Male	1.00 (ref)	-	-	1.00 (ref)	1.00 (ref)
Female	0.72 (0.45–1.16)	-	-	0.40 (0.15–1.09)	0.87 (0.48–1.56)
Marital Status					
Unmarried	1.00 (ref)	1.00 (ref)	1.00 (ref)	1.00 (ref)	1.00 (ref)
Married	1.76 (0.93–3.31)	3.04 (0.89–10.30)	1.31 (0.59–2.92)	1.07 (0.28–4.09)	1.97 (0.92–4.22)
Education					
High school or below	1.00 (ref)	1.00 (ref)	1.00 (ref)	1.00 (ref)	1.00 (ref)
Some college	1.16 (0.54–2.46)	3.28 (0.67–15.89)	0.78 (0.30–2.04)	0.23 (0.31–1.68)	1.38 (0.56–3.39)
BA	1.90 (1.07–3.37) *	1.93 (0.76–4.88)	2.07 (0.94–4.55)	1.64 (0.47–5.65)	2.07 (1.04–4.12) *
Graduate school	1.44 (0.66–3.12)	3.11 (0.84–11.43)	0.87 (0.29–2.62)	0.53 (0.12–2.43)	1.86 (0.67–5.16)
Health Insurance					
Uninsured	1.00 (ref)	1.00 (ref)	1.00 (ref)	1.00 (ref)	1.00 (ref)
Insured	0.71 (0.42–1.18)	0.85 (0.36–2.02)	0.58 (0.29–1.17)	1.65 (0.58–4.86)	0.48 (0.25–0.95) *
Member of Any Korean Association					
No	1.00 (ref)	1.00 (ref)	1.00 (ref)	1.00 (ref)	1.00 (ref)
Yes	0.89 (0.55–1.44)	1.35 (0.63–2.89)	0.52 (0.26–1.01)	0.51 (0.17–1.46)	0.93 (0.52–1.67)
Religion					
None	1.00 (ref)	1.00 (ref)	1.00 (ref)	1.00 (ref)	1.00 (ref)
Protestant	2.20 (0.79–6.11)	2.40 (0.56–10.29)	2.56 (0.49–13.46)	0.53 (0.04–5.99)	2.97 (0.85–10.39)
Catholic	1.66 (0.57–4.897)	1.69 (0.35–8.04)	1.94 (0.34–11.04)	1.86 (0.14–23.92)	1.45 (0.39–5.36)
Buddhist/other	1.74 (0.58–5.19)	2.25 (0.46–10.97)	1.23 (0.21–7.07)	0.65 (0.06–7.62)	2.39 (0.61–9.27)
Years Lived in the United States					
<10 years	1.00 (ref)	1.00 (ref)	1.00 (ref)	-	-
≥10 years	1.73 (0.99–3.04)	1.47 (0.52–4.12)	2.65 (1.24–5.66) *	-	-
English Proficiency					
Not well/a little	1.00 (ref)	1.00 (ref)	1.00 (ref)	1.00 (ref)	1.00 (ref)
Well/very well	1.23 (0.73–2.07)	1.44 (0.61–3.36)	0.91 (0.43–1.91)	1.14 (0.30–4.31)	1.07 (0.58–1.96)
Cons	2.26(0.52–9.76)	0.35 (0.03–3.58)	8.54 (0.94–76.92)	119.15 (2.13–6660) *	2.39 (0.42–13.34)
Pseudo R^2^	0.0697	0.0978	0.1392	0.2031	0.0778
*N*	421	179	242	114	307

** *p* < 0.01; * *p* < 0.05; OR, odds ratio; CI, confidence interval.
